# Role of OmpA1 and OmpA2 in *Aggregatibacter actinomycetemcomitans* and *Aggregatibacter aphrophilus* serum resistance

**DOI:** 10.1080/20002297.2018.1536192

**Published:** 2018-10-26

**Authors:** Mark Lindholm, Kyaw Min Aung, Sun Nyunt Wai, Jan Oscarsson

**Affiliations:** a Oral Microbiology, Department of Odontology, Umeå University, Umeå, Sweden; b Department of Molecular Biology and the Umeå Centre for Microbial Research (UCMR), Umeå University, Umeå, Sweden

**Keywords:** *Aggregatibacter actinomycetemcomitans*, *Aggregatibacter aphrophilus*, serum resistance, outer membrane protein A

## Abstract

*Aggregatibacter actinomycetemcomitans* and *Aggregatibacter aphrophilus* belong to the HACEK group of fastidious Gram-negative organisms, a recognized cause of infective endocarditis. *A. actinomycetemcomitans* is also implicated in aggressive forms of periodontitis. We demonstrated that *A. aphrophilus* strains, as *A. actinomycetemcomitans* are ubiquitously serum resistant. Both species encode two Outer membrane protein A paralogues, here denoted OmpA1 and OmpA2. As their respective pangenomes contain several OmpA1 and OmpA2 alleles, they represent potential genotypic markers. A naturally competent strain of *A. actinomycetemcomitans* and *A. aphrophilus*, respectively were used to elucidate if OmpA1 and OmpA2 contribute to serum resistance. Whereas OmpA1 was critical for survival of *A. actinomycetemcomitans* D7SS in 50% normal human serum (NHS), serum resistant *ompA1* mutants were fortuitously obtained, expressing enhanced levels of OmpA2. Similarly, OmpA1 rather than OmpA2 was a major contributor to serum resistance of *A. aphrophilus* HK83. Far-Western blot revealed that OmpA1^AA^, OmpA2^AA^, and OmpA1^AP^ can bind to C4-binding protein, an inhibitor of classical and mannose-binding lectin (MBL) complement activation. Indeed, *ompA1* mutants were susceptible to these pathways, but also to alternative complement activation. This may at least partly reflect a compromised outer membrane integrity but is also consistent with alternative mechanisms involved in OmpA-mediated serum resistance.

## Introduction

The HACEK group of fastidious Gram-negative organisms is a recognized cause of infective endocarditis, responsible for 1.4 to 3% of cases [1], with the genus *Aggregatibacter* now being the dominant etiology of HACEK endocarditis [2]. Colonization by the human oral bacterium, *Aggregatibacter actinomycetemcomitans* is strongly associated with aggressive forms of periodontitis in adolescents and young adults [3,4]. *Aggregatibacter aphrophilus* is a closely related organism (14.7–23.7% difference in gene content relative to *A. actinomycetemcomitans* [5]), which belongs to the indigenous human oral microflora. Infective endocarditis and cerebral abscesses are the most frequent invasive *A. aphrophilus* infections, whereas this organism is widely considered to have low virulence in periodontitis [2].

*A. actinomycetemcomitans* exhibits large genetic diversity, and serotypes form genetically divergent lineages [4]. Highly leukotoxic *A. actinomycetemcomitans* genotypes, JP2 and *cagE*, respectively (serotype b) are strongly linked to periodontal attachment loss progression in North and West African adolescents [3,6]. *A. actinomycetemcomitans* produces outer membrane vesicles (OMVs), which have been demonstrated to internalize into host cells and act as a trigger of innate immunity [7]. The systemic role of *A. actinomycetemcomitans*, in addition to its involvement in endocarditis, includes its association with cases of soft tissue abscesses, and osteomyelitis [8], and the species can be detected in atheromatous plaque [9]. It is not known if there are specific genotypes of *A. actinomycetemcomitans* (or *A. aphrophilus*) that are more prone to translocate to the peripheral circulation.

Serum resistance is an important pathogenicity determinant of Gram-negative bacteria that enter the bloodstream and cause infection, allowing their evasion of the innate defenses present in serum, including complement and antimicrobial peptides. Resistance to complement-mediated killing by human serum appears to be important for *A. actinomycetemcomitans* virulence, and it is suggested to be a common trait among strains of this species [10,11]. It is not known whether serum resistance is also frequent among *A. aphrophilus* strains. Mechanisms of bacterial resistance against complement-mediated killing include the production of protective extracellular polysaccharide capsules, and expression of factors that inhibit or interfere with the complement cascade [12]. Outer membrane integrity is important for serum resistance of Gram-negative bacteria, and in a number of species, outer membrane proteins (OMPs) have been shown to be associated with serum resistance, e.g., Ail [13], OmpW [14], PagC [15], and OmpA [16,17]. OmpA protein family members represent key components in the structural integrity of the outer membrane of bacteria and have several described pathogenic roles [18–20].

Hence, OmpA inhibition offers a strategy to combat virulence of Gram-negative organisms [21]. C4b-binding protein (C4bp) is a major inhibitor of the classical and mannose-binding lectin (MBL) pathways of the complement system [22]. Evidence has been presented that upon interacting with C4bp, *Escherichia coli* OmpA inhibits the classical complement activation cascade [23,24]. On the other hand, In *Acinetobacter baumanii*, OmpA was shown to enhance serum resistance via trapping of the alternative complement inhibitor Factor H [16].

Among the OMPs identified in *A. actinomycetemcomitans*, Omp100 (also known as ApiA) was earlier demonstrated to be important for serum resistance in some serotype b, and d strains, and to bind to the alternative pathway regulator Factor H in vitro [10,25]. Evidently, *A. actinomycetemcomitans* OMPs are immunoreactive in the human host [26]. As presence of antibodies towards OMPs is a known trigger of classical complement activation [27], serum resistance of *Aggregatibacter* spp. would be expected to include mechanisms blocking this activation. A ≈ 35-kDa, 346-amino acid heat-modifiable OmpA-like protein (also known as Omp29, and Omp34) is the most abundant *A. actinomycetemcomitans* surface protein, and a major component of outer membrane vesicles [26,28]. The *A. actinomycetemcomitans* OmpA-like protein is associated with the bacterial entry into gingival epithelial cells [29], however, its role in serum resistance has not been elucidated. Whether *A. aphrophilus* OMPs, hitherto only subjected to a preliminary characterization [30], may possibly contribute to serum resistance is not known. The aim of the present work was to investigate if OmpA proteins play a role in serum resistance in *A. actinomycetemcomitans* and *A. aphrophilus*.

## Materials and methods

### Ethics considerations

All procedures were conducted in accordance with the guidelines of the local ethics committee at the Medical Faculty of Umeå University, which are in compliance with the Declaration of Helsinki (64th WMA General Assembly, Fortaleza, October 2013). For the assays using normal human serum, blood was sampled from healthy volunteers after informed consent.

### Bacterial strains and growth conditions

*A. actinomycetemcomitans* strain D7SS is a naturally genetic competent, smooth-colony derivative of D7S (serotype a), which was originally isolated from a patient with aggressive periodontal disease [31]. Mutant *A. actinomycetemcomitans* derivatives, i.e., D7SS *ompA1::spe* [Spe^r^], D7S *ompA1::spe* [Spe^r^], D7SS *ompA2::kan* [Km^r^], D7SS *ompA1::spe, ompA2::kan* [Spe^r^, Km^r^], D7SS *omp100::kan* [Km^r^], and D7SS *omp100::kan, ompA1::spe* [Km^r^, Spe^r^] were generated in the present work. CCUG 3715 and NJ8700 are type strains of *A. aphrophilus* [32,33]. The naturally genetic competent *A. aphrophilus* strains HK83 (CCUG 49494), and CCUG 11575 were originally sampled from saliva, and a brain abscess, respectively [33]. DNA from NJ8700 was used to transform HK83 and CCUG11575 into a V factor-independent growth phenotype, following a described procedure [33]. Mutant derivatives of HK83, i.e., HK83 *ompA1::spe* [Spe^r^], HK83 *ompA2::kan* [Km^r^], and HK83 *ompA1::spe, ompA2::kan* [Spe^r^, Km^r^] were generated in the present work. Strains AHI-3151, IH-90256, and IH-90274 are part of the collection of clinical isolates of *A. aphrophilus* in our laboratory, established by Dr. Sirkka Asikainen. The *A. aphrophilus* strains 4 Aap-K, 12 Aap-K, 13 Aap-K, 21 Aap-K, 29 Aap-K, 30 Aap-K, 32 Aap-K, and 53 Aap-K belong to our bacterial strain collection at the clinical laboratory, Oral Microbiology. The *A. actinomycetemcomitans* and *A. aphrophilus* strains were routinely cultivated in air supplemented with 5% CO_2_, at 37°C, on blood agar plates (5% defibrinated horse blood, 5 mg hemin/l, 10 mg Vitamin K/l, Columbia agar base). Alternatively, for transformation assays, the strains were grown on Trypticase soy broth supplemented with 0.1% yeast extract, 5% heat-inactivated horse serum, and 1.5% agar (sTSB agar), and when needed, supplemented with 100 μg/ml (final concentration) spectinomycin, or kanamycin. *E. coli* K-12 laboratory strain DH5α was used for maintenance of plasmids and was cultured aerobically at 37°C in Luria-Bertani (LB) broth, or on LB broth solidified with 1.5% (w/v) agar.

### *Construction of outer membrane protein gene replacement mutants in* A. actinomycetemcomitans *and* A. aphrophilus

A PCR-based approach following standard cloning procedures was used to construct gene replacement mutants in naturally competent strains of *A. actinomycetemcomitans* (D7SS), and *A. aphrophilus* (HK83). For *A. actinomycetemcomitans*, the strain D7S-1 complete genome (GenBank accession CP003496) was used as reference in oligonucleotide synthesis. For *A. aphrophilus*, strain NJ8700 was used as reference genome (GenBank accession CP001607). Whole genome sequence data of the *A. aphrophilus* model strain HK83 was kindly communicated by Niels Nørskov-Lauritsen and has been deposited at DDBJ/ENA/GenBank under the accession QMGS00000000. Sequences of the oligonucleotide primers used for mutant construction and outer membrane protein gene database entries are listed in Table 1. In brief, PCR fragments flanking the target genes were amplified. The PCR primers contained BamHI or SalI restriction sites, allowing ligation of the PCR fragments to flank either the spectinomycin resistance gene from plasmid pK-Spe [34], or the kanamycin resistance gene from pUC4K [35]. Ligation products were then used to transform D7SS and HK83 on agar plates using procedures described earlier [31]. Confirmation of allelic replacements and the orientation of the inserted resistance cassette were done by DNA sequencing and PCR. For this we used a target gene-specific F1, and R2 oligonucleotide primer (Table 1), respectively in combination with a primer specific for the appropriate antibiotic determinant, i.e., spectinomycin (Spe1: 5ʹ-CCACTCTCAACTCCTGATCC-3ʹ) or kanamycin (H7R: 5ʹ- GGACGGCGGCTTTGTTGAATAAATCG-3ʹ).

**Table 1.** Target *ompA1* and *ompA2* genes in the *A. actinomycetemcomitans* and *A. aphrophilus* reference strains D7S-1 and NJ8700, respectively, and oligonucleotide primers used for the generation of allelic replacement mutants.

Target gene GenBank accession number, and definition in database	Oligonucleotide^a^ (F: forward; R: reverse)	PCR product length (bp)^b^
**Primers for *A. actinomycetemcomitans***		
*ompA1^AA c^* AFI86243 ‘membrane protein’	F1: 5ʹ- CTGCTTCACAAATAAAGGCGAGGGAG-3ʹ R1: 5ʹ- GATTGCAGTT**GTCGAC**ATTTTGATGATCC-3ʹ F2: 5ʹ-GAAATCGCT**GTCGAC**GGTACTAAATA-3ʹ R2: 5ʹ-GCTCACGGCGGCAATCAATAAC-3’	1,295 1,287
*ompA2^AA d^* AFI86283 ‘membrane protein’	F1: 5ʹ-GAATCCCACGTCACCGTGCC-3ʹ R1: 5ʹ-GTAGCTAATG**GGATCC**CAGTTTTTTTC-3ʹ F2: 5ʹ-GTGACAC**GGATCC**AGGTCGCAAAG-3ʹ R2: 5ʹ-CCGACAGTGGAATGTACGAAAACTAC-3ʹ	1,269 1,061
*omp100^e^* AFI 86,288 ‘membrane protein’	F1: 5ʹ-GCATGGCGTCCAATAAACCTTG-3ʹ R1: 5ʹ-GTTTAAATAAT**GGATCC**GTCATAATTCA-3ʹ F2: 5ʹ-CTATAACGTC**GGATCC**AACTTTGAGTG-3ʹ R2: 5ʹ-GCATGGTTGGAACGCTTCTTACAC-3ʹ	1,386 1,208
**Primers for *A. aphrophilus***		
*ompA1^AP f^* ACS96969 ‘outer membrane protein A’	F1: 5ʹ-GTCGGATTTGACCGCACTTGTGTC-3ʹ R1: 5ʹ-GATAGCTAATGCGA**GTCGAC**TTTTTTTCA-3ʹ F2: 5ʹ-GTAGAAATCGCT**GTCGAC**GGTAGCAA −3ʹ R2: 5ʹ- GAG AAT CGG GAA AGG TCA CGG CT-3’	1,081 1,280
*ompA2^AP g^* ACS97183 ‘outer membrane protein A’	F1: 5ʹ-GGGAGCAGAGTGAGCAGGTG-3ʹ R1: 5ʹ-CAGCTAATG**GGATCC**TAGTTTTTTTC F2: 5ʹ-GAAATTGCAGTAAATGG**GGATCC**ATAATT-3ʹ R2: 5ʹ-CAACTGACTCAACTCATCGAACAG-3’	1,328 1,322

^a^Primers introducing BamHI (GGATCC) and SalI (GTCGAC) restriction sites (sequences in underlined bold) as indicated.

^b^Predicted based on genome sequences of D7S-1 and NJ8700, respectively.

^c^Also referred to as Omp29, Omp34, and OmpA-like protein.

^d^Encoded protein exhibits ≈ 76% amino acid identity with OmpA1^AA^

^e^Also referred to as ApiA.

^f^Encoded protein exhibits ≈ 83% amino acid identity with OmpA1^AA^

^g^Encoded protein exhibits ≈ 71% amino acid identity with OmpA1^AA^

### SDS-PAGE and Western blot analysis

The procedures used for SDS-PAGE and Western blot analysis have been described previously [36]. Gels were stained using Coomassie Brilliant Blue R-250 (Bio-Rad) or Pierce® Silver Stain Kit (Thermo Fisher Scientific). Selected protein bands after Coomassie- or Silver-staining were excised from gels and subject to liquid chromatography tandem mass spectrometry (LC-MS/MS) at the Proteomics Core Facility at Chemical Biological Centre, Umeå University and Swedish University of Agricultural Sciences. For Western blot, we used a rabbit polyclonal antiserum specific for *E. coli* OmpA [37] (final dilution 1:10,000), and a patient serum from a periodontitis subject, which exhibits strong reactivity to *A. actinomycetemcomitans* antigens [38] (1:2,000). As secondary antibody, anti-rabbit or anti-human horseradish peroxidase (HRP)-conjugate was used (Jackson ImmunoResearch, Newmarket, UK) (1:10,000). Immunoreactive bands were visualized using Clarity™ Western ECL Substrate (Bio-Rad) or SuperSignal® West Pico Chemiluminescent Substrate (Thermo Fisher Scientific), and the ChemiDoc™ XRS + System (Bio-Rad).

### Far-Western blot analysis

To detect binding of the complement regulator, C4b-binding protein to OmpA, the immunoblot membrane was blocked in tris-buffered saline, 0.1% tween 20 (TBS-T) with milk (5% w/v) overnight at 4°C, and then incubated with 1 μg/ml (final dilution) human recombinant C4bp (Abcam) in TBS-T with milk (0.5% w/v) for 2 h at room temperature. After washing four times with TBS-T, the membrane was incubated with a rabbit polyclonal antiserum specific for human C4bp (Abcam) (final dilution 1:1,500). After four washes with TBS-T, anti-rabbit HRP-conjugate (Jackson ImmunoResearch, Newmarket, UK) was used at 1:10,000, and immune detection was then performed as described above.

### Extraction of outer membrane proteins

Outer membrane protein bacterial fractions were isolated essentially as described earlier [36]. *A. actinomycetemcomitans* or *A. aphrophilus* cells were harvested in 10 mM HEPES (pH 7.4) from an average of four blood agar plates. After centrifugation (40,000 × *g*; 20 min, 4°C), bacterial pellets were resuspended in HEPES and subject to sonication (30-second pulses; 30% amplitude). After removal of intact cells and cell debris (1,700 × *g*; 20 min, 4°C), supernatants were subject to ultracentrifugation (100,000 × *g*; 1 h, 4°C) using an SW60Ti rotor (Beckman Instruments Inc.). Pellets were resuspended in 2% (w/v) sodium-lauryl-sarcosinate (Sigma-Aldrich) in HEPES, and after sequential steps of ultracentrifugation (100,000 × *g*; 1 h, 4°C; SW60Ti) pellets were resuspended in buffer A (1% [w/v] n-octyl-β-D-glucopyranoside [Sigma-Aldrich] in 50 mM Tris, 5 mM EDTA, pH 8.0), in buffer A containing 0.5M NaCl, and finally in 20 mM sodium phosphate buffer (pH 7.5) containing 0.1% (w/v) SDS.

### Isolation of outer membrane vesicles

As an alternative approach to obtain bacterial fractions enriched in OmpA proteins, OMVs were isolated from *A. actinomycetemcomitans* and *A. aphrophilus* cells harvested from an average of 10 blood agar plates, using ultracentrifugation as described earlier [7]. OMV pellets were washed twice with PBS (85,000 × *g*; 2 h, 4°C) using a 70 Ti rotor (Beckman Instruments Inc.), and then used as the OMV preparations. The yield of OMVs was estimated by quantifying vesicle preparations for protein content using a NanoDrop 1000 spectrophotometer (Thermo Fisher Scientific). To assess the uniformity of OMV preparations, samples were validated by atomic force microscopy (AFM), and SDS-PAGE. OMVs were also checked for absence of bacterial contamination by cultivating small aliquots on blood agar plates in air supplemented with 5% CO_2_, at 37°C for 3 days.

### Atomic force microscopy

For AFM, bacterial cells or OMV preparations were diluted with ultrapure water (Millipore) and placed onto a freshly cleaved mica surface. Samples were incubated for 5 min at room temperature, washed with ultrapure water, and then placed in a desiccator for ~2 h in order to dry. The samples were finally magnified through a Nanoscope V Atomic Force Microscope (Bruker AXS GmbH, Karlsruhe, Germany), using tapping mode. Final images were plane fitted in both the *x* and *y* axes and are presented in amplitude mode.

### Electron microscopy

Analyses were carried out at the Umeå Core Facility for Electron Microscopy (UCEM). For transmission electron microscopy of OMV preparations, 3.5 µl of samples was applied to glow discharged formvar and carbon coated Cu-grids. The grids were washed and negatively stained in 1.5% Uranyl acetate for 2 × 15 sec. Samples were examined with a Talos 120C transmission electron microscope (FEI, Eindhoven, The Netherlands) operating at 120 kV. Micrographs were acquired with a Ceta 16M CCD camera (FEI, Eindhoven, The Netherlands) using TEM Image & Analysis software version 4.14 (FEI, Eindhoven, The Netherlands). For scanning electron microscopy, small pieces of agar containing bacterial colonies were fixed in 2.5% glutaraldehyde in 0.1 M sodium cacodylate buffer (pH 7.4) at 4°C overnight, then dehydrated in graded series of ethanol, critical point dried, and metal-coated (4 nm). The morphology of the samples was analyzed with a field-emission scanning electron microscope (Zeiss Merlin FESEM).

### Bacterial serum killing assay

To monitor the sensitivity of bacterial strains to normal human serum (NHS), NHS was taken from healthy volunteers. We largely followed procedures described previously [10,27], using *A. actinomycetemcomitans*, and *A. aphrophilus* strains grown on agar. In brief, prior to being used in the assays, bacteria were harvested and suspensions were adjusted to 1.0 × 10^9^ cells/ml in PBS. Reaction mixtures contained 105 μl NHS, 95 μl PBS, and 10 μl bacterial suspension and were incubated at 37°C for 2 h. Cell survival was assessed by plating serial dilutions on agar. Heat-inactivated (56°C, 30 min) NHS (HI-NHS) was used as control. Survival rates were calculated as the ratio (%) of colony forming units (CFU) NHS/HI-NHS. When needed to distinguish between the classical and MBL, and the alternative pathway of complement activation, serum killing assays were performed in the presence of 5 mM MgCl_2_, and 10 mM EGTA (Mg^2+^/EGTA), which selectively permits the alternative pathway only [39]. To obtain NHS depleted from the MBL pathway, serum was filtered through D-mannose-agarose beads (Sigma-Aldrich, St. Louis, USA), as described previously [27].

### Statistical analysis and image processing

The statistical significance of the data was calculated using two-tailed Student’s t-test. The level of statistical significance was set to *P *< 0.05, based on at least three independent experiments unless otherwise stated. Data are expressed as means ± standard errors of the means (SEM). Images for figures were assembled using Adobe Photoshop CS6 or Microsoft PowerPoint.

## Results

### Both *A. actinomycetemcomitans* and *A. aphrophilus* encode two OmpA proteins

To assess the prevalence of *ompA* genes in these species, we screened all whole genome sequences of *A. actinomycetemcomitans* strains (n = 38) and *A. aphrophilus* (n = 7) available in the National Center for Biotechnology (NCBI) database in September 2017. The result of this *in silico* analysis is summarized in Supplementary Table 1. Of the *A. actinomycetemcomitans* strains, 37 were found to encode the major OmpA protein (in the present work referred to as OmpA1^AA^). Strain SA3733 (serotype d), encoded a partial OmpA1 sequence. In total, six OmpA1^AA^ alleles were identified exhibiting 99–100% amino acid identity to the strain D7S protein. A 356-amino acid protein sharing approximately 76% amino acid identity with OmpA1^AA^ is in this work referred to as OmpA2^AA^. The database searches revealed OmpA2^AA^ to be encoded by 37 out of the 38 *A. actinomycetemcomitans* genomes (not identified only in SA3733). In total, six OmpA2^AA^ alleles were identified exhibiting 97–100% amino acid identity to the strain D7S protein. As there were allele combinations specific for some serotypes, e.g., OmpA1^AA^-4 and OmpA2^AA^-4 in serotype b, these gene and protein sequences may represent potential genotyping markers. According to the database searches, *A. aphrophilus*, similar to *A. actinomycetemcomitans* encodes two OmpA paralogues. These proteins (346 and 363–366 amino acids, respectively) exhibit ≈ 74% amino acid identity, and we have in this study referred to them as OmpA1^AP^ and OmpA2^AP^. OmpA1^AP^ was encoded in all *A. aphrophilus* genomes, and in total three alleles were identified exhibiting 96–100% amino acid identity to the strain NJ8700 protein. A gene encoding OmpA2^AP^ was identified in all *A. aphrophilus* genomes, except ATCC7901 and in total five alleles were identified exhibiting 96–100% amino acid identity to the strain NJ8700 protein. Thus, a large majority of *A. actinomycetemcomitans* and *A. aphrophilus* strains encode two OmpA proteins.

### OmpA1 is important for serum resistance in *A. actinomycetemcomitans* strains D7SS and D7S

**Figure 1.**
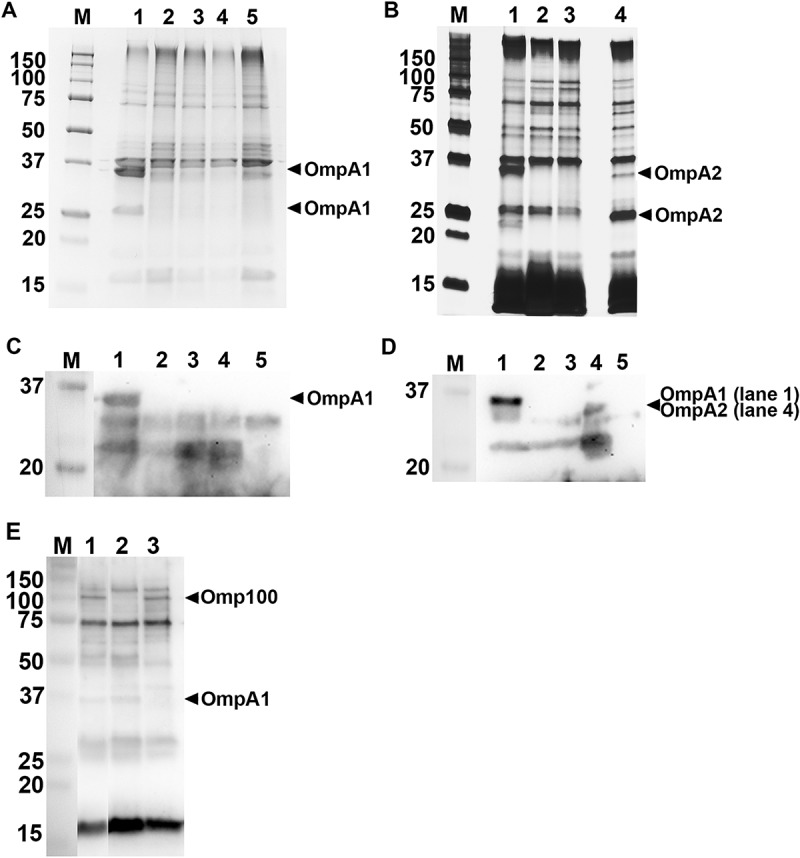
SDS-PAGE analysis of outer membrane protein, and outer membrane vesicle preparations obtained from *A. actinomycetemcomitans* strains. OMP preparations were analyzed using Coomassie-staining (a), and Western blot using a polyclonal antiserum specific for *E. coli* OmpA (c). OMV preparations were analyzed using Silver-staining (b), and Western blot using a polyclonal antiserum specific for *E. coli* OmpA (d), or an *A. actinomycetemcomitans*-responsive serum from a periodontitis subject (e). Samples from the following strains are shown in the panels: D7SS (lane 1), D7SS *omp100 ompA1* (lane 2 in panel a–d), D7SS *ompA1* (lane 3), D7SS *ompA1* R1 (lane 4), D7SS *ompA1 ompA2* (lane 5), and D7SS *omp100* (lane 2 in panel e). Selected protein bands are indicated with arrows. Sizes (kDa) of the proteins in the pre-stained molecular weight marker (M) are indicated along the left side.

**Figure 2.**
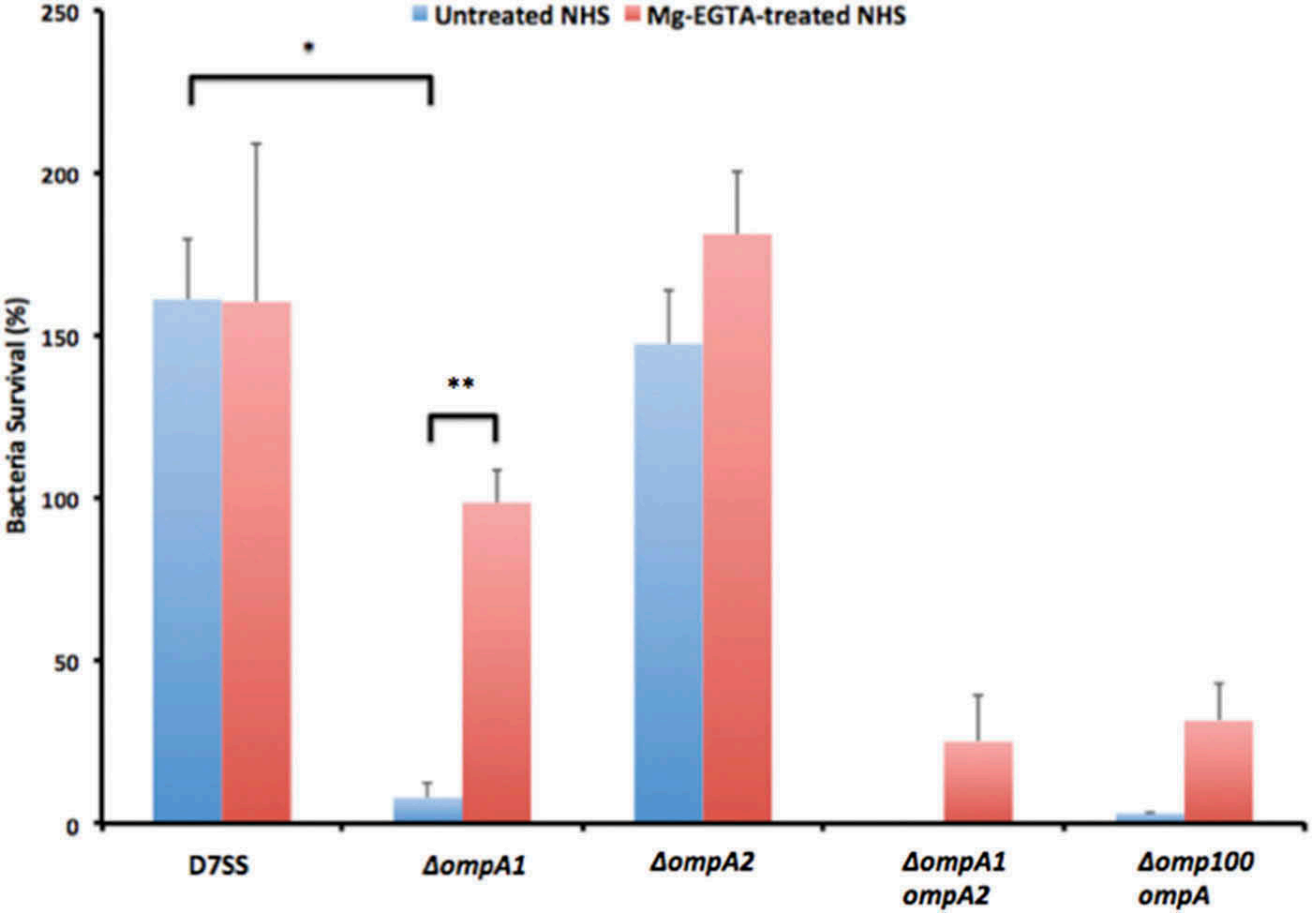
Analysis of the serum survival of *A. actinomycetemcomitans* strain D7SS and its mutant derivatives. 1.0 × 10^9^ bacterial cells were incubated in 50% normal human serum (NHS), or 50% heat-inactivated (HI)-NHS at 37°C for 2 h. The assay was performed in the absence (blue bars) or presence (red bars) of Mg^2+^/EGTA. Bacterial serum survival was determined by viable count and expressed as ratio (%) of CFU in NHS/HI-NHS. Shown are means ± SEM from at least three independent experiments, with the exception of the D7SS *ompA1 ompA2* double mutant, which was assessed twice. *P < 0.001, D7SS vs. D7SS *ompA1* in the absence of Mg^2+^/EGTA; **P < 0.001, D7SS *ompA1* in the absence vs. presence of Mg^2+^/EGTA.

To investigate if OmpA1^AA^ contributes to serum resistance, the *ompA1^AA^* gene was subject to allelic replacement in strains D7S and D7SS. The abolished expression of OmpA1 in the mutants was confirmed by SDS-PAGE and Western blot, analyzing OMP (Figure 1(a,c)), and OMV preparations (Figure 1(b, d, and e)), respectively. These analyses revealed distinct protein bands at approximately 35 and 25 kDa, respectively, which were also confirmed to represent OmpA1^AA^ using LC-MS/MS, and which were absent in the *ompA1* mutant derivatives. According to serum killing assays using 50% NHS (Figure 2), strain D7SS was completely serum resistant (survival rate determined to 161.3%) whereas its *ompA1* mutant derivative exhibited high susceptibility (survival rate 8.0%). Essentially similar survival rates were obtained when assessing the rough-colony D7SS parental strain, D7S (161.4% ± 21.9% [SEM]), and its *ompA1* mutant derivative (1.1% ± 0.9% [SEM]). In contrast to the observations with *ompA1*, inactivation of *omp100* in strain D7SS did not result in a low survival rate (106.6% ± 19.8% [SEM]). Lack of Omp100 production in the mutant was confirmed by Western blot (Figure 1(e)). Consistent with a major role of OmpA1 in serum resistance, inactivation of the *ompA1 *gene in the D7SS *omp100* mutant led to a severely reduced survival rate, 3.1% (Figure 2). Based on these results together, we concluded that OmpA1 was a major contributor to the serum resistance of *A. actinomycetemcomitans* D7SS, and its parental strain D7S.

### A. actinomycetemcomitans strain *D7SS* ompA1 *mutant derivatives regained serum resistance upon enhanced production of OmpA2*

**Figure 3.**
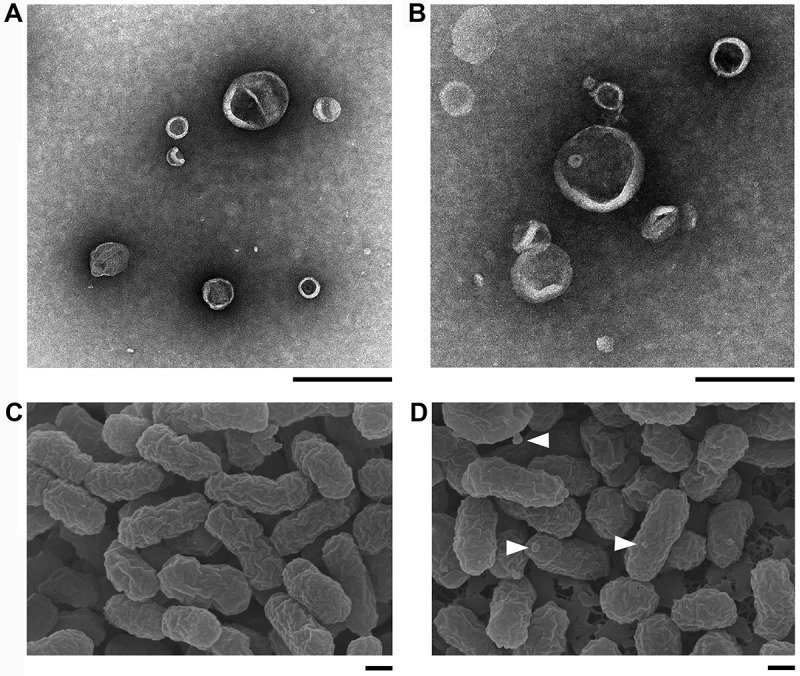
Electron micrographs of *A. actinomycetemcomitans* strains and their released OMVs. Transmission electron micrographs of OMVs released from D7SS (a), and D7SS *ompA1 ompA2* (b). Scanning electron micrographs of D7SS (c), and D7SS *ompA1 ompA2* (d) cultivated on agar. The arrows in panel d indicate examples of the larger membrane vesicle-like structures occasionally found projecting from the cell surface of the double mutant. Bars = 200 μm.

We made attempts to clone *ompA1*^AA^ behind the leukotoxin promoter using the pVT1503 *A. actinomycetemcomitans* shuttle vector, and the same approach as for the complementation of *bilRI* mutants [40]. Unfortunately, this resulted solely in clones with a truncated C-terminus (data not shown), suggesting that overexpression of OmpA1 was toxic to the *E. coli* laboratory strain used. Nonetheless, colonies of the D7SS *ompA1^AA^* mutant were fortuitously obtained after the incubation in 50% NHS. After isolation of such clones from independent experiments, they were found to exhibit a serum survival rate, comparable to D7SS. Interestingly, according to SDS-PAGE analysis of OMV preparations from one selected serum resistant *ompA1 *mutant clone, denoted D7SS *ompA1*-R1 (survival rate 186.5% ± 62.1% [SEM]), there were two enhanced protein bands at approximate sizes very similar to OmpA1, i.e. ≈ 35 and ≈ 25 kDa (Figure 1(b,d)). These proteins, which appeared to be produced at a low level in the serum-sensitive D7SS *ompA1* mutant derivative, were subject to LC-MS/MS analysis. This revealed that both protein bands corresponded to OmpA2^AA^. Thus, we concluded that the protein identified in the lower molecular weight band likely represented a degradation product of full length OmpA2. To investigate if serum resistant D7SS *ompA1* derivatives may represent a heterogeneous population of mutants, OMV preparations from five additional clones were analyzed by SDS-PAGE (Supplementary Figure 1). This revealed upregulated OmpA2 production in four of the tested clones, suggesting that this is a common characteristic among D7SS *ompA1* mutants surviving in 50% NHS. The role of OmpA2 was evaluated in D7SS *ompA1*, and in D7SS *ompA1*-R1, by allelic replacement of *ompA2^AA^*. As judged by the very low to non-existing serum survival rates of the double mutants, D7SS *ompA1 ompA2* (0%), and D7SS *ompA1*-R1 *ompA2* (1.8% ± 0.6% [SEM]), OmpA2 indeed contributed to serum resistance. In contrast, allelic replacement of *ompA2^AA^* in an *ompA1^AA^*^+^ gene background did not result in an apparent decrease in serum resistance (Figure 2). The survival rates of the wild-type strain and its mutant derivatives were similar in heat-inactivated 50% NHS (data not shown), whereas there was a somewhat reduced growth rate of the D7SS *ompA1* single and *ompA1 ompA2* double mutants in liquid cultures (Supplementary Figure 2). Electron microscopy revealed that OMV preparations from both D7SS and D7SS *ompA1 ompA2* contained vesicles of various sizes (diameter approximately 30–200 nm) (Figure 3(a-b)). However, larger membrane vesicle-like structures were occasionally observed projecting from the cell surface of the double mutant, which was not observed assessing the wild-type (Figure 3(c-d)). This suggests that the serum sensitivity was accompanied by at least partially impaired membrane stability. Based on these results together, we concluded that in the absence of OmpA1, serum resistance of *A. actinomycetemcomitans* strain D7SS could be regained via increased production of OmpA2. As judged by DNA sequencing, background mechanisms why OmpA2 appeared to be produced at enhanced levels in the serum resistant D7SS *ompA1 *mutant clones did not include alterations in the *ompA2* promoter and upstream region, as amplified using the primers *ompA2^AA^* F1 and *ompA2^AA^* R1 (data not shown).

### *Serum resistance in* A. aphrophilus *strains*

Prompted by our findings with *A. actinomycetemcomitans*, we wanted to investigate if OmpA proteins may contribute to serum resistance in an additional species of this genus, i.e., the close relative *A. aphrophilus*. In serum killing assays using 50% NHS, we screened our available (n = 15) *A. aphrophilus* strains in our collections, observing that they displayed moderate to high levels of serum resistance (Supplementary Table 2). Albeit this initial screening suggested that the degrees of serum resistance may vary among strains, it supported the notion that serum resistance may be ubiquitous also among *A. aphrophilus* strains. Strain HK83 was selected for genetic analysis as it is a naturally competent strain, facilitating the genetic analysis.

### OmpA protein expression in *A. aphrophilus* strain HK83

**Figure 4.**
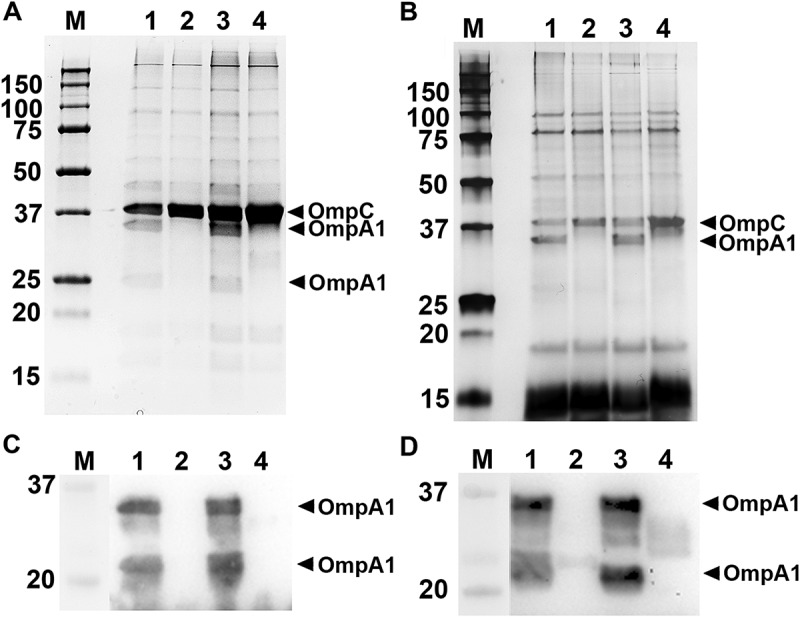
SDS-PAGE analysis of outer membrane protein, and outer membrane vesicle preparations obtained from *A. aphrophilus* strains. OMP preparations were analyzed with Coomassie-staining (a), and Western blot using a polyclonal antiserum specific for *E. coli* OmpA (c). OMV preparations were analyzed with Silver-staining (b), and Western blot using a polyclonal antiserum specific for *E. coli* OmpA (d). Samples from the following strains are shown in each panel: HK83 (lane 1), HK83 *ompA1* (lane 2), HK83 *ompA2* (lane 3), and HK83 *ompA1 ompA2* (lane 4). Selected protein bands are indicated with arrows. Sizes (kDa) of the proteins in the pre-stained molecular weight marker (M) are indicated along the left side.

**Figure 5.**
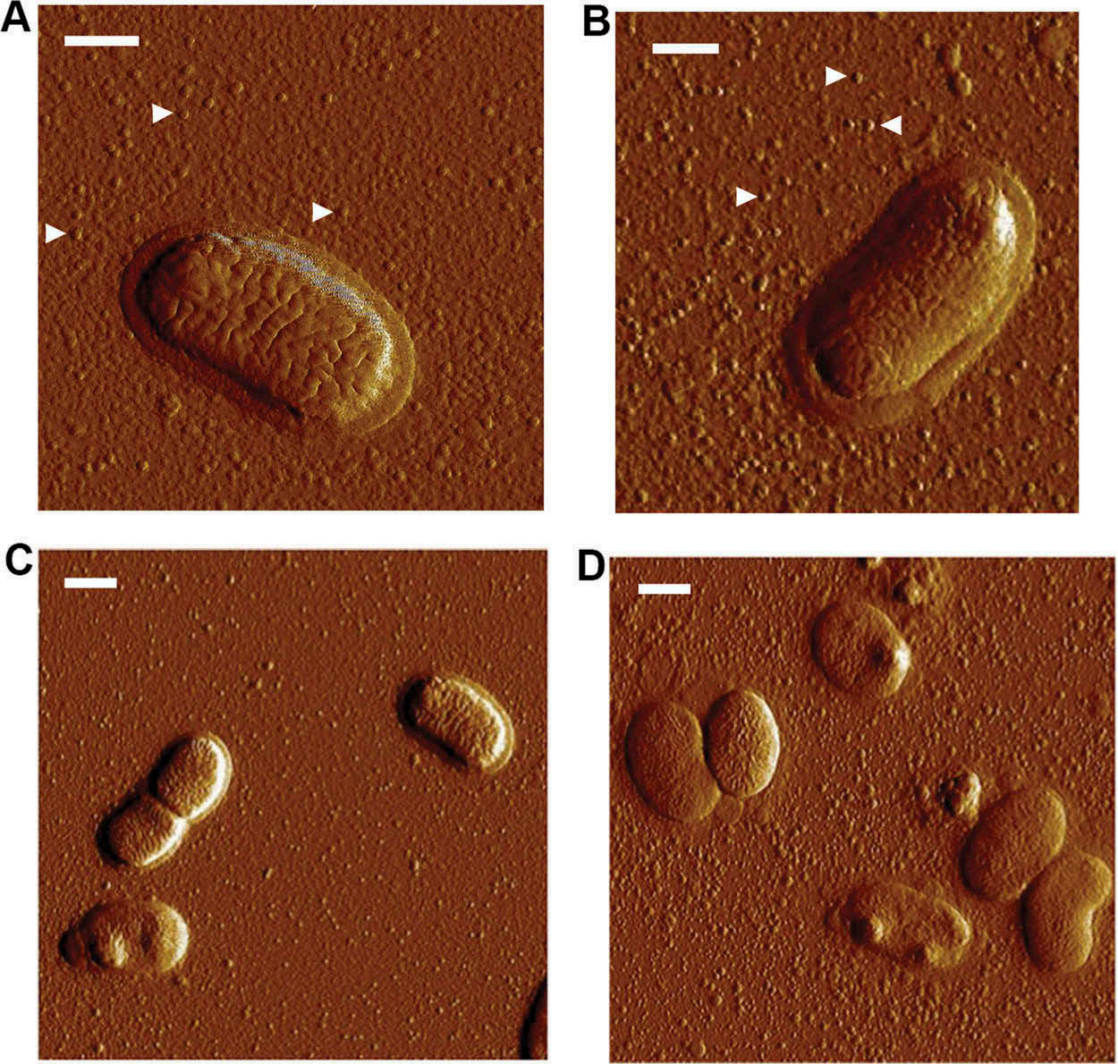
Release of OMVs from *A. aphrophilus* strains. Atomic force micrographs of bacterial strains cultivated on agar. *A. aphrophilus* strains HK83 (a and c), CCUG 3715 (b), and HK83 *ompA1 ompA2* (d), which produces large numbers of OMVs relative to HK83. Arrows indicate examples of the released *A. aphrophilus* vesicles in a and b. Bars = 300 nm (a, b), and 500 nm (c, d).

Analysis of whole genome sequencing data of *A. aphrophilus* strain HK83 confirmed that it encodes OmpA1^AP^ and OmpA2^AP^, both sharing approximately 96% amino acid identity with the corresponding proteins of the *A. aphrophilus* reference strain NJ8700. We noted that the OmpA1^AP^ protein sequence of HK83 is identical to that of *A. aphrophilus* strains ATCC 7901 and W10433 (accession numbers OBY53818 and AKU63521, respectively), and that the HK83 OmpA2^AP^ protein is identical to that of strain W10433 (accession number AKU63719). Strain HK83 was used to generate *ompA1*^AP^ and *ompA2^AP^* single, and *ompA1^AP^ ompA2^AP^* double mutants, respectively. To estimate the relative levels of produced OmpA1^AP^ and OmpA2^AP^ in HK83, SDS-PAGE and Western blot were used to analyze OMP preparations (Figure 4(a,c)). In addition, OMV preparations were assessed (Figure 4(b,d)) as *A. aphrophilus* strains were found to produce outer membrane vesicles, at a level similar to *A. actinomycetemcomitans* (Figure 5). These analyses revealed distinct protein bands at approximately 35 and 25 kDa, respectively, which were confirmed to represent OmpA1^AP^ using LC-MS/MS, and which were absent in the *ompA1^AP^* mutant derivatives (Figure 4). This observation is consistent with the notion that OmpA1^AP^ was the major OmpA protein produced under the growth conditions used. Moreover, SDS-PAGE revealed two protein bands with somewhat increased intensity, at approximately 37 kDa in OMP and OMV preparations from the HK83 *ompA1*^AP^*ompA2*^AP^ double mutant (Figure 4(a,b)). According to LC-MS/MS analysis of these protein bands, the slightly larger-sized protein was identified as OmpC (AKU63902), a homologue to *A. actinomycetemcomitans* Omp39 (≈ 73% amino acid identity). This may represent compensatory expression of OmpC due to lack of OmpA1^AP^ and OmpA2^AP^. The second protein band was identified as a methyl-galactoside ABC transporter substrate-binding protein (OBY51871).

### OmpA1 is important for the serum resistance of *A. aphrophilus* strain HK83

**Figure 6.**
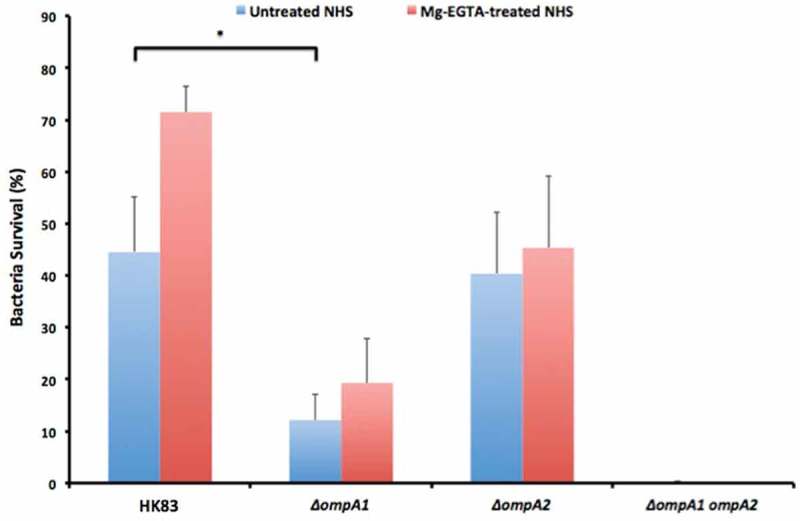
Analysis of the serum survival of *A. aphrophilus* strain HK83 and its mutant derivatives. 1.0 × 10^9^ bacterial cells were incubated in 50% normal human serum (NHS), or 50% heat-inactivated (HI)-NHS at 37°C for 2 h. The assay was performed in the absence (blue bars) or presence (red bars) of Mg^2+^/EGTA. Bacterial serum survival was determined by viable count and expressed as ratio (%) of CFU in NHS/HI-NHS. Shown are means ± SEM from at least three independent experiments. *P < 0.04, HK83 vs HK83 *ompA1* for both conditions.

To elucidate if OmpA1^AP^ and/or OmpA2^AP^ might be involved in *A. aphrophilus* serum resistance, strain HK83 and its *ompA1* and *ompA2* single, and *ompA1 ompA2 *double mutants were assessed in serum killing experiments using 50% NHS. The *ompA1* mutant exhibited a survival rate of 12.1%, i.e. almost four times lower than the parental strain, HK83 (Figure 6), clearly supporting that OmpA1^AP^ was contributing to serum resistance. Similar to the findings with *A. actinomycetemcomitans* strain D7SS, inactivation of *ompA2*^AP^ in HK83 did not significantly affect the serum survival. However, serum resistance was almost abolished in the *ompA1 ompA2* double mutant (Figure 6), indicating that OmpA2^AP^ contributed to the serum resistance in this background. The survival rates of the wild-type strain and its mutant derivatives were similar in heat-inactivated NHS (data not shown), whereas like the *A. actinomycetemcomitans* strains, there was a somewhat reduced growth rate of the HK83 *ompA1* single and *ompA1 ompA2* double mutants in liquid cultures (Supplementary Figure 2). As judged by the hypervesiculation phenotype of HK83 *ompA1 ompA2* revealed by AFM (Figure 5(d)), its serum sensitivity was most likely accompanied by a disruption of the outer membrane integrity. Analogously to our findings with the *A*. *actinomycetemcomitans* strain, clones of the HK83 *ompA1* mutant were fortuitously obtained after the incubation in 50% NHS, which after isolation were found to exhibit serum resistance at a level comparable to the parental strain. As judged by SDS-PAGE analysis of OMV preparations from selected *ompA1* mutant clones with enhanced serum resistance, there was no noticeable difference in the levels of outer membrane proteins relative to the parental strain (data not shown). One clone with enhanced serum resistance, HK83 *ompA1*-R1 (survival rate 56.1% ± 12.6% [SEM]), was subject to inactivation of *ompA2*^AP^. As this resulted in essentially abolished serum survival (< 1%), we concluded that OmpA2 contributed to the serum resistance in this background.

### Complement activation pathways effective in elimination of serum-sensitive *OmpA* mutant derivatives

To initially investigate mechanisms for OmpA-mediated serum resistance, we assessed which pathways of complement activation were effective in elimination of serum-sensitive *ompA1* single and *ompA1 ompA2* double mutant strains of the two species. To this end, serum killing assays were performed in the presence of Mg^2+^/EGTA, which allow alternative complement activation but inhibits the classical and MBL pathways (Figure 2). Whereas blocking the classical and MBL pathways had no effect on survival of the serum resistant *A. actinomycetemcomitans* strain D7SS, its *ompA1 *mutant displayed a largely increased survival rate (66.0%). Comparably, in the presence of Mg^2+^/EGTA there was much enhanced survival rates of the D7SS *ompA1 ompA2* (25.3%), and *ompA1 omp100* (31.7%), double mutants, respectively, and in serum-killing assays using MBL-depleted NHS, the survival rate of the D7SS *ompA1* mutant was relatively high, i.e. 77.9% ± 27.6% [SEM]). This together supports that the serum susceptibility of the *ompA1*^AA^ mutant derivatives to a relatively large extent was mediated via classical/MBL complement activation. However, as in the presence of Mg^2+^/EGTA, none of the serum-sensitive D7SS mutant derivatives exhibited a survival rate corresponding to that of their respective parental strains (Figure 2), we concluded that they were also susceptible to alternative complement activation.

In the presence of Mg^2+^/EGTA the serum survival rate of the *A. aphrophilus* wild-type strain, HK83, and its *ompA2* derivative was 71.5% and 45.3%, respectively, consistent with a partial susceptibility to both classical/MBL and alternative complement activation (Figure 6). Likewise, although there was enhanced survival of the HK83 *ompA1* mutant in the presence of Mg^2+^/EGTA, its survival rate was still as low as 19.3%, whereas the HK83 *ompA1 ompA2* double mutant was essentially serum-sensitive. Moreover, the survival rate of the HK83 *ompA1* mutant was enhanced when MBL-depleted serum was used (39.3% ± 13.9% [SEM]). Hence, taken together, these results support the notion that the classical, MBL, and alternative complement activation pathways were all involved in the elimination of the serum-sensitive *ompA* mutant derivatives tested. This suggests that alternate mechanisms may be involved in OmpA-mediated serum resistance.

### OmpA-dependent trapping of C4b binding protein (C4bp)

**Figure 7.**
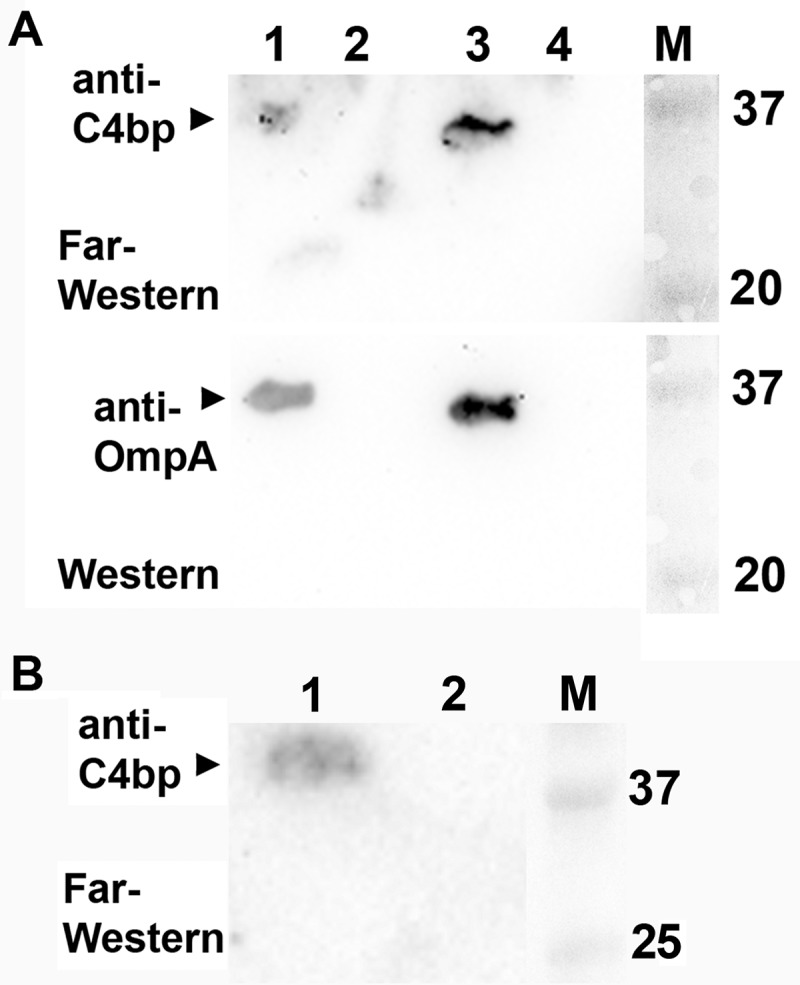
Far-Western analysis to detect binding of OmpA1^AA^ and OmpA1^AP^ to C4b-binding protein (C4bp). (a) OMVs obtained from *A. actinomycetemcomitans* D7SS (lane 1), and D7SS *ompA1* (lane 2), and from *A. aphrophilus* HK83 (lane 3), and HK83 *ompA1* (lane 4) were subjected to SDS-PAGE. For Far-Western analysis, the membrane was first incubated with C4bp (1 μg/ml final dilution) prior to immunodetection using an anti-C4bp polyclonal antibody. OmpA was detected using a polyclonal antiserum specific for *E. coli* OmpA. (b) Far-Western analysis of OMVs obtained from *A. actinomycetemcomitans* D7SS *ompA1*-R1 (lane 1), and D7SS *ompA1* (lane 2), respectively. Sizes (kDa) of the proteins in the pre-stained molecular weight marker (M) are indicated along the right side.

To investigate possible mechanisms how the *Aggregatibacter* OmpA proteins may protect the bacterial cells from complement-mediated killing, we investigated whether OmpA1^AA^ and OmpA1^AP^, similar to *E. coli* OmpA [24], might be able to bind to C4bp. For this, OMVs obtained from *A. actinomycetemcomitans* D7SS and *A. aphrophilus* HK83, and their respective *ompA1* mutants, were assessed by Far-Western analysis using recombinant human C4bp, and polyclonal anti-C4bp, and anti-OmpA antibodies, respectively. The detection using the anti-C4bp antibody indicated that there was binding of C4bp as represented by a protein band on the membrane at a position corresponding to the size of OmpA, which was observed in the case of both wild-type strains (Figure 7(a)). This observation was confirmed using the anti-OmpA antibody (Figure 7(a)). In contrast, there was no such C4bp binding detected assessing the OMV samples from the *ompA1^AA^* and *ompA1^AP^* mutant, respectively (Figure 7(a)). Moreover, also OmpA2^AA^ was found to bind to C4bp, assessing OMVs from the serum resistant strain D7SS *ompA1*-R1 (Figure 7(b)). These results together are consistent with the notion that both *A. actinomycetemcomitans* and *A. aphrophilus* can exhibit OmpA-dependent binding of C4bp.

## Discussion

In the present work we have used naturally competent model strains of *A. actinomycetemcomitans* (D7SS; serotype a), and its close relative *A. aphrophilus* (HK83) to demonstrate the role of OmpA proteins in serum resistance of these organisms.

The observation that most whole genome-sequenced *A. actinomycetemcomitans* and *A. aphrophilus* strains encode two OmpA paralogues is in accordance with findings with a number of other Gram-negative species, e.g., *Aeromonas salmonicida, Bacteroides fragilis, Haemophilus ducreyi*, and *Porphyromonas gingivalis* [19,41–43]. We have referred to the major (i.e., the most abundant) OmpA protein of *A. actinomycetemcomitans* and *A. aphrophilus* as OmpA1^AA^ and OmpA1^AP^, respectively, for consistence with studies on the other organisms encoding two OmpA paralogues. The observation that the pangenomes of these two species encode several alleles of OmpA1 and OmpA2, respectively, suggests that they represent potential genotypic markers, similar to the *ompA* sequences in *Chlamydia trachomatis* and *Pasteurella multocida* [44,45].

Based on previous studies with *A. actinomycetemcomitans* [10,46], we typically assessed the serum survival in 50% NHS. The observation that *A. aphrophilus* strains, similar to *A. actinomycetemcomitans* are ubiquitously resistant to killing by normal human serum is consistent with their association with extra-oral infections such as infective endocarditis and cerebral abscesses [2]. A role of OmpA proteins in serum resistance of *A. actinomycetemcomitans* and *A. aphrophilus* is in agreement with findings with a number of other bacterial species, including *A. baumanii* and *E. coli* [16,17]. In both *Aggregatibacter* species tested in the present work, the major OmpA protein, OmpA1 was important for the serum survival, albeit not essential, as a low frequency of the *ompA1*^AA^ and *ompA1*^AP^ mutant derivatives survived in the presence of 50% NHS. The interesting finding that *ompA1*^AA^ mutants fortuitously regained their serum resistance upon increased OmpA2^AA^ production, supported that OmpA2^AA^ here acted as a functional homologue to OmpA1^AA^, which is consistent with the high degree of amino acid identity of these proteins, and with previous observations of compensatory OMP expression upon loss of major outer membrane porins [47]. Similarly, our present work suggests that there was compensatory production of OmpC and/or ABC transporter substrate protein in the *ompA1*^AP^*ompA2*^AP^ double mutant, which would be in accordance with the role of homologues to these proteins in bacterial serum resistance [48,49].

The finding that no clones could be obtained expressing the full length OmpA1^AA^ protein for mutant complementation, but rather a C-terminally truncated form, implies that overexpression of OmpA1 may be deleterious to the *E. coli* laboratory strain used. The phenomenon that OmpA of some bacterial species can be toxic in *E. coli* strains has earlier been described e.g., with OmpA of *Haemophilus influenzae, Mycobacterium tuberculosis*, and *Rhodopseudomonas blastica* [50–52]. In line with the essentially abolished serum survival of the *ompA1 ompA2* double mutants of both species, EM and AFM, respectively revealed that they exhibited larger membrane-vesicle like structures projecting from the cell surface, and/or a hypervesiculation phenotype, consistent with earlier observations with *ompA* mutants exhibiting impaired membrane stability [19,53–56], and reduced growth [57].

In this work, we have also attempted to investigate putative mechanism(s) for OmpA-mediated protection of the bacterial cells from complement-mediated killing. Similar to earlier findings with *Klebsiella pneumoniae* [58], we observed that several complement activation pathways appeared to be effective in the elimination of serum-sensitive *ompA1* single, and *ompA1 ompA2* double mutant derivatives of *A. actinomycetemcomitans* and *A. aphrophilus*. This may, at least partly reflect a compromised outer membrane integrity in these mutants, which would be consistent with their somewhat reduced growth rates, observed in liquid cultures. However, evidently, OmpA proteins have been demonstrated to interact with serum components that regulate both the classical/MBL, and alternative pathways of complement activation, respectively [16,23,24]. Our finding that *A. actinomycetemcomitans* OmpA1 and OmpA2, and *A. aphrophilus* OmpA1 could bind to the classical/MBL pathway negative regulator, C4bp is consistent with observations with *E. coli* OmpA. It remains, however to be elucidated to which extent this interaction per se plays an active role in conferring serum resistance, similar to what was demonstrated with *E. coli* OmpA [23,24]. In contrast to earlier findings with a couple of *A. actinomycetemcomitans* serotype b and d strains [10,25], inactivation of the *omp100* gene locus in the serotype a strain D7SS did not render the cells serum-susceptible. The reason for this discrepancy is not known but may reflect the relative levels of produced OMPs in the different strains under the conditions used. Such a scenario is plausible as judged by observations with *Salmonella* Enteritidis that the patterns of OMP expression vary among strains exhibiting different degrees of serum sensitivity [59]. The notion that *A. actinomycetemcomitans* and *A. aphrophilus* may exploit alternate mechanisms to evade complement-mediated killing is supported by recent findings that strains display different patterns of complement activation pathway component consumption in vitro [60]. Moreover, it has been demonstrated that human serum induces global stress responses to varying degrees in *A. actinomycetemcomitans* strains [46]. Thus, it is likely that serum resistance in both *Aggregatibacter* species, similar to *A. baumanii* [48], is highly complex, relying on a large number of gene products.

In summary, the present work has revealed the importance of primarily OmpA1, but also OmpA2 in serum resistance of *A. actinomycetemcomitans* and *A. aphrophilus*. The recognition of bacterial products mediating serum resistance represents an approach to vaccine and drug development. The existence of different alleles of OmpA1 and OmpA2 among strains in these species can possibly be utilized in the development of strain-specific vaccines and agents that reduce the serum resistance.
