# Supplementary material for: Role of OmpA1 and OmpA2 in Aggregatibacter actinomycetemcomitans and Aggregatibacter aphrophilus serum resistance
Source: J Oral Microbiol. 2018 Oct 26;11(1):1536192. doi: 10.1080/20002297.2018.1536192 (PMC6225413; doi:10.1080/20002297.2018.1536192)
Supplement: Supplemental Material [file ZJOM_A_1536192_SM3976.docx]

**Supplemental material**

**
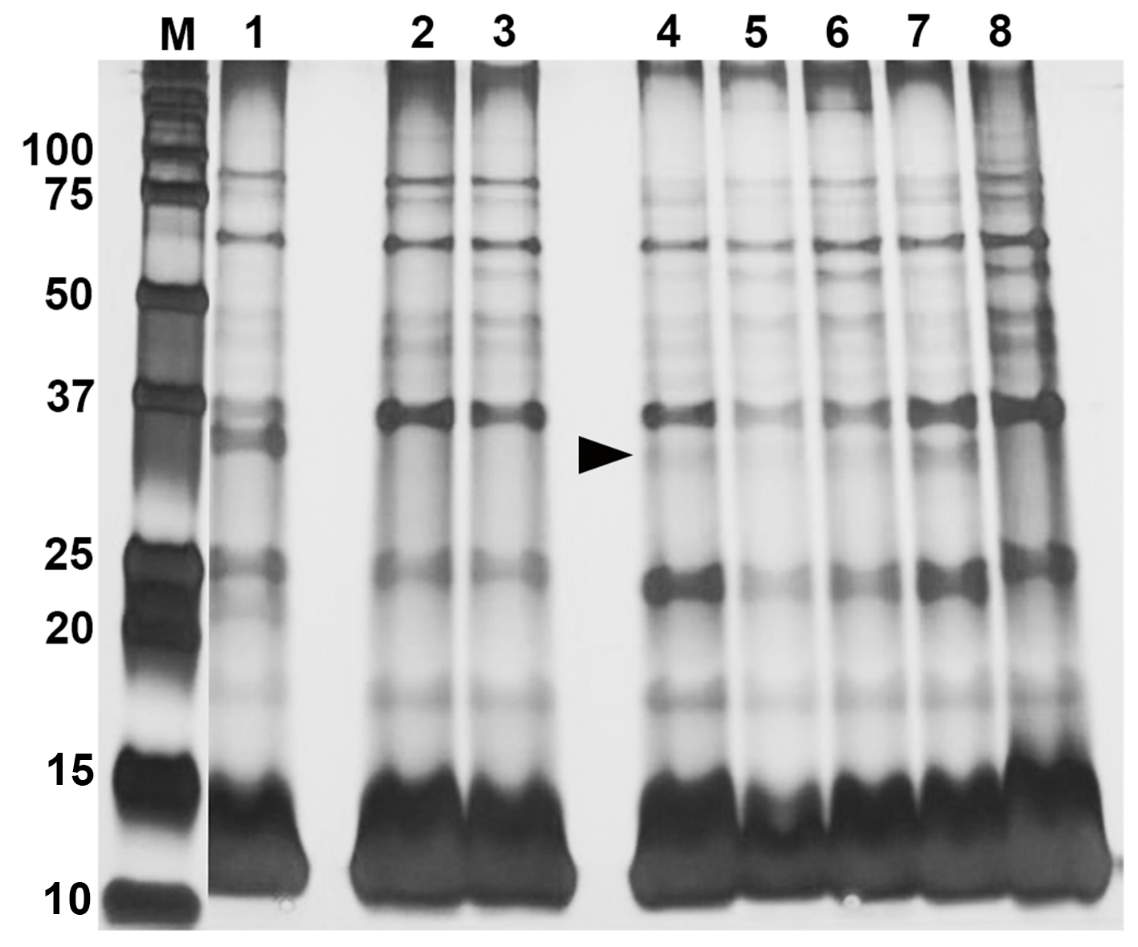
**

**Supplementary Figure 1.** SDS-PAGE analysis of outer membrane vesicle preparations obtained from *A. actinomycetemcomitans* strains. OMV preparations were analyzed with Silver-staining. Samples from the following strains were loaded on the gel: D7SS (lane 1), D7SS *ompA1* (serum sensitive; lane 2 and 3), serum resistant clones of D7SS *ompA1* (R2-R6; lanes 4-8). The protein bands corresponding to OmpA2 is indicating with an arrow. Sizes (kDa) of the proteins in the prestained molecular weight marker (M) are indicated along the left side.

**
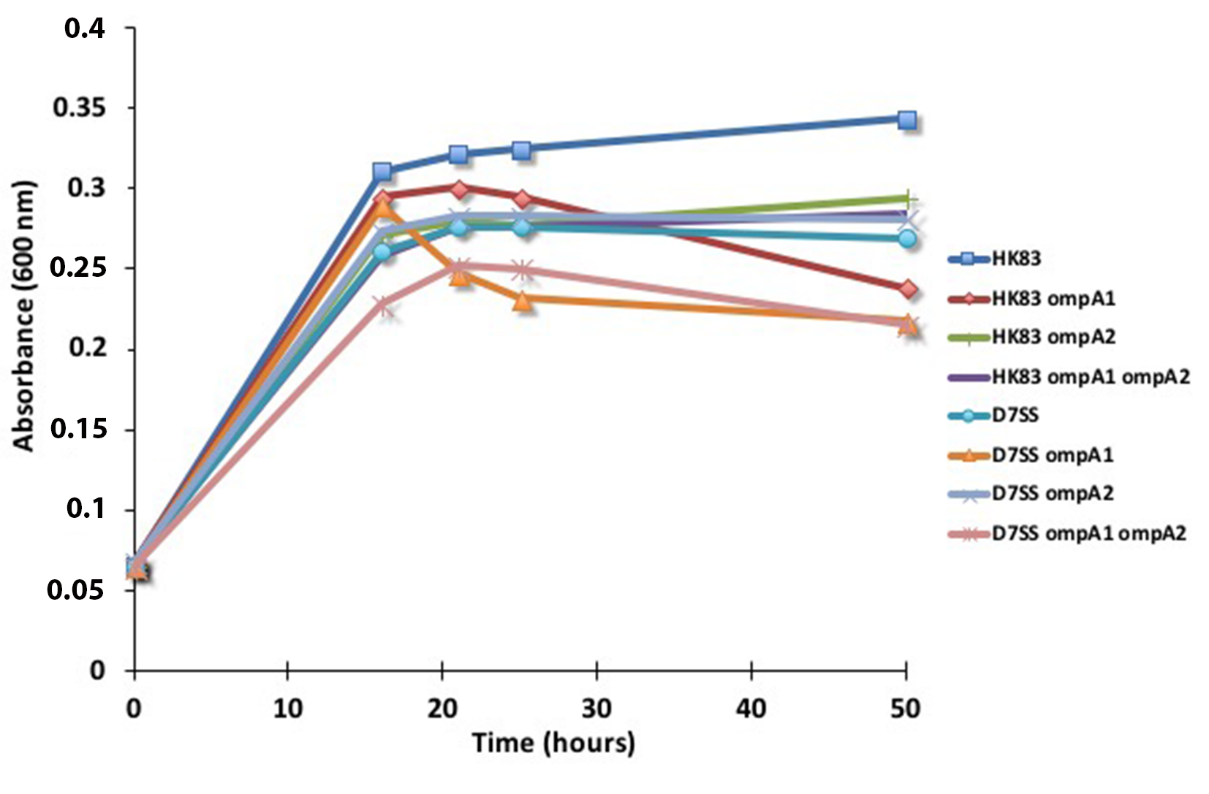
**

**Supplementary Figure 2.** Growth rates of *A. actinomycetemcomitans* and *A. aphrophilus* strains D7SS and HK83, and their respective *ompA1* and *ompA2* mutant derivatives. The strains were cultured in TSB broth and growth was monitored for 2 d.

| **OmpA1 protein sequences in *A. actinomycetemcomitans* genomes.** | | | | | |
| --- | --- | --- | --- | --- | --- |
| OmpA1^AA^  type | Number of  amino acids | GenBank  accession  number | Identified in genome  (serotype) | Name in  database | Amino acid  identity  to  OmpA1 in D7S |
| 1 | 346 | AFI86243  AMQ93302  ANU82584  BAS48718  EKX98206  KOE30751  KND85496 KOE63422 KYK73043 KYK90815 KYK84536 KYK94433 KYK90225 PHO22207  PHO20144 | D7S (a)  624 (a)  IDH781 (d)  NUM4039 (g)  Y4 (b)  D17-P3 (a)  H5P1 (a) I63B (d) SA2149 (e) SA508 (d) SA3033 (d) SA269 (d) SA2200 (d) 310a 310b | Membrane protein  Hypothetical protein  Hypothetical protein  OmpA protein  Putative outer membrane protein A  Membrane protein  Membrane protein  Membrane protein  Membrane protein  Membrane protein  Membrane protein  Membrane protein  Membrane protein  Porin OmpA  Porin OmpA | 100% |
| 2 | 346 | AAC00068  ACX82938 KOE59243 KOE66369 KOE66983 KYK79721 KYK80295 KYK88952 OZV16827 N/A | NCTC 9710 (c) D11S-1 (c) D17P-2 (c) SCC393 (e) A160 (a) SA2876 (e) SC383s (c) SC29R (f) KCOM 1299 DSM 8324 | Outer membrane protein 34  Membrane protein  Membrane protein  Membrane protein  Membrane protein  Membrane protein  Membrane protein  Membrane protein  Membrane protein  Porin OmpA | 99% |
| 3 | 346 | EHK89959 | RhAA1 | Outer membrane protein A | 99% |
| 4 | 346 | AEW77844  AHN72573  AMQ91930  BAA75215 KND83138 KOE52909 KOE56470 KOE57070 | ANH9381 (b)  HK1651 (b)  VT1169 (b)  Y4 (b)  SCC1398 (b) I23C (b) SCC4092 (b) S23A (b) | OmpA-like outer membrane protein  Outer membrane protein,OmpA family  Hypothetical protein  OmpA-like outer membrane protein  Membrane protein  Membrane protein  Membrane protein  Membrane protein | 99% |
| 5 | 346 | EGY32758  KYK76545  KYK79776  KYK95989 | SC1083 (e)  SA3096 (e)  SC936 (e)  ANH9776 (e) | OmpA-like outer membrane protein  Membrane protein  Membrane protein  Membrane protein | 99% |
| 6 | 345 | KOE58090  KOE60544  KOE69854 | AAS4A (c)  SCC2302 (c)  D18P1 (f) | Membrane protein  Membrane protein  Membrane protein | 99% |

| **OmpA2 protein sequences in *A. actinomycetemcomitans* genomes.** | | | | | |
| --- | --- | --- | --- | --- | --- |
| OmpA2^AA^  type | Number of  amino acids | GenBank  accession  number | Identified in genome  (serotype) | Name in  database | Amino acid  identity  to OmpA2 in D7S |
| 1 | 356 | AFI86283  AMQ93336  ANU82619 BAS48680  EKX94942 KND85096  KOE30483  KOE63762  KOE64651  KOE65520  KYK75832  KYK84934  KYK86236  KYK89575  KYK90275  KYK90801  OZV16861  PHO21384  PHO23618 | D7S (a)  624 (a)  IDH781 (d) NUM4039 (g) Y4 (b) H5P1 (a)  D17-P3 (a)  A160 (a)  SCC393 (e)  I63B (d)  SA2876 (e)  SA3033 (d)  SC29R (f)  SA2200 (d)  SA508 (d)  SA269 (d)  KCOM1299  310b  310a | Membrane protein  Hypothetical protein  Hypothetical protein  Outer membrane protein A  Putative outer membrane protein A  Membrane protein  Membrane protein  Membrane protein  Membrane protein  Membrane protein  Membrane protein  Membrane protein  Membrane protein  Membrane protein  Membrane protein  Membrane protein  Porin OmpA  Porin OmpA  Porin OmpA | 100% |
| 2 | 356 | ACX82953 KOE58075 KOE60559 KOE61947 KYK76472 KYK80310 N/A | D11S-1 (c) AAS4A (c) SCC2302 (c) D17P-2 (c) SA2149 (e) SC383s (c) DSM 8324 | Membrane protein  Membrane protein  Membrane protein  Membrane protein  Membrane protein  Membrane protein  Porin OmpA | 99% |
| 3 | 356 | KOE69990 | D18P1 (f) | Membrane protein | 99% |
| 4 | 356 | AEW77859  AHN72591  AMQ91943 KND83124 KOE52924 KOE56484 KOE57085 | ANH9381 (b)  HK1651 (b)  VT1169 (b)  SCC1398 (b) I23C (b) SCC4092 (b) S23A (b) | Outer membrane protein A  Outer membrane protein,OmpA family  Hypothetical protein  Membrane protein  Membrane protein  Membrane protein  Membrane protein | 99% |
| 5 | 356 | EHK89767 | RhAA1 | Outer membrane protein A | 97% |
| 6 | 356 | EGY34295  KYK72633  KYK78565  KYK91635 | SC1083 (e)  SA3096 (e)  SC936 (e)  ANH9776 (e) | Outer membrane protein A  Membrane protein  Membrane protein  Membrane protein | 97% |

| **OmpA1 protein sequences in *A. aphrophilus* genomes.** | | | | | |
| --- | --- | --- | --- | --- | --- |
| OmpA1^AP^  type | Number of  amino acids | GenBank  accession  number | Identified in genome | Name in  database | Amino acid  identity  to OmpA1 in NJ8700 |
| 1 | 346 | ACS96969  EHB91136  PNL93723 | NJ8700  F0387  FDAARGOS_248 | Outer membrane protein A  Hypothetical protein  Porin OmpA | 100% |
| 2 | 346 | KNE85909  OBY49874 | ATCC 33389  ATCC 19415 | Membrane protein  Hypothetical protein | 98% |
| 3 | 346 | AKU63521  OBY53818  N/A | W10433  ATCC 7901  HK83 | Hypothetical protein  Hypothetical protein  N/A | 96% |

| **OmpA2 protein sequences in *A. aphrophilus* genomes.** | | | | | |
| --- | --- | --- | --- | --- | --- |
| OmpA2^AP^  type | Number of  amino acids | GenBank  accession  number | Identified in genome | Name in  database | Amino acid  identity  to OmpA2 in NJ8700 |
| 1 | 363 | ACS97183 | NJ8700 | Outer membrane protein A | 100% |
| 2 | 363 | PNL93521 | FDAARGOS_248 | Porin OmpA | 99% |
| 3 | 363 | EHB90571 | F0387 | Hypothetical protein | 99% |
| 4 | 363 | KNE86128  OBY55313 | ATCC 33389  ATCC 19415 | Membrane protein  Hypothetical protein | 99% |
| 5 | 366 | AKU63719  N/A | W10433  HK83 | Hypothetical protein  N/A | 96% |

**Supplementary Table 1.** OmpA1 and OmpA2 protein sequences in *A. actinomycetemcomitans* and *A. aphrophilus* strains according to *in silico* analysis of available genome databases (National Center for Biotechnology [NCBI]). D7S and NJ8700, respectively have been used as reference genomes. N/A = not available.

| Strain | Serum survival (%, NHS/HI-NHS) |
| --- | --- |
| AHI-3151 | 86 |
| CCUG 11575 | 72 |
| CCUG 3715 | 96 |
| HK83 | 44.5 ± 10.5% [SEM] |
| IH-90256 | 132 |
| IH-90274 | 178 |
| NJ8700 | 267 |
| 4 Aap-K | 314 |
| 12 Aap-K | 134 |
| 13 Aap-K | 159 |
| 21 Aap-K | 298 |
| 29 Aap-K | 152 |
| 30 Aap-K | 61 |
| 32 Aap-K | 394 |
| 53 Aap-K | 240 |

**Supplementary Table 2.** *A. aphrophilus* strains are ubiquitously serum resistant. A collection of *A. aphrophilus* strains was screened for levels of serum survival. 1.0 × 10^9^ bacterial cells were incubated in 50% normal human serum (NHS), or 50% heat-inactivated (HI)-NHS at 37°C for 2 h. Bacterial serum survival was determined by viable count and expressed as ratio (%) of colony forming units (CFU) in NHS/HI-NHS.
